# Rurality and patients’ hospital experience: A multisite analysis from a US healthcare system

**DOI:** 10.1371/journal.pone.0308564

**Published:** 2024-08-08

**Authors:** Iman Fawad, Karen M. Fischer, Hanieh Sadat Tabatabaei Yeganeh, Kristine T. Hanson, Laurie L. Wilshusen, Yousif M. Hydoub, Trevor J. Coons, Tafi L. Vista, Michael J. Maniaci, Elizabeth B. Habermann, Sagar B. Dugani

**Affiliations:** 1 Division of Hospital Internal Medicine, Mayo Clinic, Rochester, Minnesota, United States of America; 2 Department of Quantitative Health Sciences, Mayo Clinic, Rochester, Minnesota, United States of America; 3 Division of Endocrinology, Diabetes and Metabolism, Mayo Clinic, Rochester, Minnesota, United States of America; 4 Robert D. and Patricia E. Kern Center for the Science of Health Care Delivery, Mayo Clinic, Rochester, Minnesota, United States of America; 5 Mayo Clinic Quality, Mayo Clinic, Phoenix, Arizona, United States of America; 6 Division of Cardiology, Sheikh Shakhbout Medical City, Abu Dhabi, United Arab Emirates; 7 Division of Hospital Internal Medicine, Mayo Clinic, Jacksonville, Florida, United States of America; Bahir Dar University, ETHIOPIA

## Abstract

**Background:**

The association between rurality of patients’ residence and hospital experience is incompletely described. The objective of the study was to compare hospital experience by rurality of patients’ residence.

**Methods:**

From a US Midwest institution’s 17 hospitals, we included 56,685 patients who returned a post-hospital Hospital Consumer Assessment of Healthcare Providers and Systems (HCAHPS) survey. We defined rurality using rural-urban commuting area codes (metropolitan, micropolitan, small town, rural). We evaluated the association of patient characteristics with top-box score (favorable response) for 10 HCAHPS items (six composite, two individual, two global). We obtained adjusted odds ratios (aOR [95% CI]) from logistic regression models including patient characteristics. We used key driver analysis to identify associations between HCAHPS items and global rating (combined *overall rating of hospital* and *recommend hospital*).

**Results:**

Of all items, *overall rating of hospital* had lower odds of favorable response for patients from metropolitan (0.88 [0.81–0.94]), micropolitan (0.86 [0.79–0.94]), and small towns (0.90 [0.82–0.98]) compared with rural areas (global test, *P* = .003). For five items, lower odds of favorable response was observed for select areas compared with rural; for example, *recommend hospital* for patients from micropolitan (0.88 [0.81–0.97]) but not metropolitan (0.97 [0.89–1.05]) or small towns (0.93 [0.85–1.02]). For four items, rurality showed no association. In metropolitan, micropolitan, and small towns, men vs. women had higher odds of favorable response to most items, whereas in rural areas, sex-based differences were largely absent. Key driver analysis identified *care transition*, *communication about medicines* and *environment* as drivers of global rating, independent of rurality.

**Conclusions:**

Rural patients reported similar or modestly more favorable hospital experience. Determinants of favorable experience across rurality categories may inform system-wide and targeted improvement.

## Introduction

Patients’ hospital experience is associated with adherence to recommendations, clinical outcomes, and health care resource utilization [1–7]. While rural, compared with nonrural, patients experience disparities in care and outcomes, [8–12] improved understanding of their hospital experience may guide targeted improvement.

Overall, there is sparse information on the association of rurality and patient experience. Based on US hospital-level data from 2006–2007, hospitals in rural areas had significantly higher scores for most measures of Hospital Consumer Assessment of Healthcare Providers and Systems (HCHAPS), [13] a national survey of patients’ hospital experience that facilitates standardized evaluation and improvement of patient experience [14]. In a multilevel analysis of hospitals and patients in Massachusetts assessing three *satisfaction* measures (staff responsiveness, hospital cleanliness, hospital quietness), rural vs. urban patients had lower likelihood of satisfaction with staff responsiveness [11]. There are few non-US studies on rurality and patient experience. In a recent study, rural Chinese residents reported a better experience with *healthcare services* when compared to urban residents [15]. This single-item question reflected experience with hospitals and clinic services [15]. Scottish residents in rural and remote areas, compared to other areas, reported higher *satisfaction* with patient-centered care and continuity of care provided by general practitioners [16]. These studies provided insight from diverse settings and using select measures of hospital experience. However, to our knowledge, there are no studies on how rural residence impacts diverse measures of hospital experience, including the determinants and drivers of hospital experience.

To better understand and characterize the hospital experience of rural patients, we leveraged routinely collected patient-level HCAPHS survey data available for 56,685 patients at an institution’s 17 hospitals in two US states from 2016–2019. We compared hospital experience by rurality of patients’ residence and evaluated demographic and hospital characteristics in order to identify drivers of patient experience across rurality categories and guide improvement in patient experience.

## Materials and methods

### Study design and participants

Our Hospital Internal Medicine Patient Reported Outcomes Versus Experience (Hospital IMPROVE) initiative evaluates patient experience to guide improvement. The design, data source, and participants have been described [17]. Briefly, in this retrospective cross-sectional study, participants were patients who returned the HCAHPS survey following hospitalization on a medical or non-obstetric surgical service at Mayo Clinic Rochester or Mayo Clinic Health System (MCHS) in Minnesota and Wisconsin. MCHS is a network including 16 hospitals in Minnesota and Wisconsin organized into four regions: southeast Minnesota (SEMN; Austin/Albert Lea, Cannon Falls, Lake City, Red Wing); southwest Minnesota (SWMN; Fairmont, Mankato, New Prague, St. James, Waseca); northwest Wisconsin (NWWI; Barron, Bloomer, Eau Claire, Menomonie, Osseo); and southwest Wisconsin (SWWI; La Crosse, Sparta). Mayo Clinic hospitals in Florida and Arizona were excluded because most survey respondents resided in metropolitan areas, precluding analysis by rurality. We excluded patients with obstetric admissions because they are frequently elective, of shorter duration, and with routine post-hospital follow-up, compared to patients from medical or surgical service lines. Data for individual study sites were not reported to protect site confidentiality. This analysis was based on 56,685 unique surveys (i.e., first available survey per patient) returned between January 1, 2016, and December 31, 2019. The analysis was completed from September 1, 2022 to March 1, 2024.

### Sampling process and HCAHPS survey instrument

Following hospitalization, patients received the HCAHPS survey from a third-party vendor (Press Ganey) in English, Spanish, or Arabic, based on their language preference in the electronic health record. Patients did not receive the survey if (i) aged <18 years at hospital admission, (ii) not hospitalized overnight, (iii) in legal custody, (iv) lacked a US home address, (v) discharged to another facility, or (vi) on a “do not survey” list. For patients with multiple surveys, only the first survey was included. The response rate was 25%–32% per quarter; non-respondents were not characterized due to unavailable data, as described [18]. For this analysis, we excluded participants who did not provide authorization for inclusion in research studies.

HCAHPS included composite measures, individual items, and global items [19]. The composite measures were *communication with nurses* with 3 sub-items, *communication with doctors* with 3 sub-items, *responsiveness of hospital staff* with 2 sub-items, *communication about medicines* with 2 sub-items, *discharge information* with 2 sub-items, and *care transition* with 3 sub-items; individual items were *cleanliness of the hospital environment* and *quietness of hospital environment*; and, global items were *overall rating of hospital* and *recommend hospital*. Two composite measures (*responsiveness of hospital staff* and *communication about medicines*) had branching logic with responses for ~67% of patients. Individual questions that constituted *pain management* (composite measure) changed during the study period and were not evaluated. An item was categorized as ‘favorable’ (i.e., ‘top-box’) if the response was ‘always’, ‘yes’, ‘definitely yes’, ‘strongly agree’, or score of 9 or 10. All other non-missing responses were categorized as ‘unfavorable’ (i.e., ‘non–top-box’). If all items within a composite measure had a ‘favorable’ response, then the composite measure was categorized as ‘favorable’ [18].

### Patient and hospitalization characteristics

We extracted data on patient and hospitalization characteristics from the Mayo Clinic Unified Data Platform, which comprises pooled electronic medical record data [17].

### Primary outcome

The primary outcome was favorable patient experience for each of the six composite measures, two individual items, and two global items.

### Rurality

Rurality of patients’ residence was defined using 2010 Rural-Urban Commuting Area (RUCA) codes described by the US Department of Agriculture [20]. RUCA codes classify census tracts based on population density, urbanization, and daily commuting, and are based on data from the 2010 decennial census and 2006–10 American Community Survey [20]. The RUCA classification includes 10 primary codes and 21 secondary codes to classify rurality. Residence zip codes were matched with RUCA code and categorized as metropolitan (codes 1−3), micropolitan (codes 4−6), small town (codes 7−9) or rural (code 10) [20]. In this classification system, the proportion of rural respondents in different sites was 12.3% in Rochester (N = 4782/38,751), 13.2% in SEMN (N = 396/3000), 18.2% in SWMN (N = 942/5170), 18.5% in NWWI (N = 1273/6867), and 13.8% in SWWI (N = 400/2897).

### Statistical analysis

We used descriptive statistics to compare patient characteristics and experience across rurality categories with Kruskal-Wallis (continuous variables) and Chi-square (categorical variables) tests. Within each rurality category (metropolitan, micropolitan, small town, and rural), we used separate multivariable logistic regression models to evaluate the associations between patient characteristics and favorable patient experience (outcome). To compare associations across rurality categories, we included the entire cohort in a mixed model logistic regression model evaluating associations between rurality categories (reference: rural) and favorable patient experience (outcome). The models were adjusted for age, sex (men, women), race (White, other/unknown), service line (medical, surgical), hospital length of stay, and Elixhauser comorbidity index [21] (tertiles) as fixed effects. Study site (Rochester, NWWI, SWWI, SEMN, SWMN) was used as a random effect. Model results were reported as the adjusted odds ratio (aOR) and 95% confidence interval. The key driver analysis is described [18]. Briefly, we plotted the mean of each composite measure (linear scale) against the Spearman correlation coefficient of that measure with the global score (linear scale) to identify the association between composite and global scores. We analyzed data using SAS^®^ 9.4 (SAS Institute Inc.) with statistical significance at 2-tailed *P* < .05. For multiple comparison, Bonferroni correction (*P* < .005) was used, as indicated in the Table footnote.

### Ethics approval

This study was deemed exempt by the Mayo Clinic institutional review board. The use of secondary data did not require written or verbal consent. The study statistician and principal investigator had access to information that could identify participants.

## Results

### Baseline characteristics

Among 56,685 patients including 48.8% women (N = 27,682/56,685) and 95.3% of White race (N = 54,036/56,685), most resided in metropolitan (51.7%; N = 29,322), followed by micropolitan (18.6%; N = 10,536), small town (15.9%, N = 9034), and rural (13.7%; N = 7793) areas (Table 1). Across rurality categories, patients differed statistically in all the examined demographic and hospitalization characteristics. The proportion of women ranged from 47.2% (rural) to 49.2% (metropolitan) (*P* = .02), the proportion with White race ranged from 94.4% (metropolitan) to 96.5% (small town) (*P* < .0001), and the proportion discharged from the medical service line ranged from 35.2% (metropolitan) to 41.8% (small town) (*P* < .0001).

**Table 1:** Patient and hospitalization characteristics based on rurality of patients’ residence.

	Rurality of Patients’ Residence				
	Metropolitan N = 29,322	Micropolitan N = 10,536	Small town N = 9034	Rural N = 7793	*P* value
Age, years	67 (18–104)	69 (18–104)	70 (18–102)	69 (18–100)	< .0001
Women	14,412 (49.2)	5165 (49.0)	4424 (49.0)	3681 (47.2)	.02
Ethnicity					< .0001
Hispanic or Latino^a^	167 (0.6)	35 (0.3)	34 (0.4)	24 (0.3)	
Non-Hispanic or Latino	11,685 (39.9)	4295 (40.8)	3877 (42.9)	3175 (40.7)	
Unknown	17,470 (59.6)	6206 (58.9)	5123 (56.7)	4594 (59.0)	
Race					< .0001
American Indian or Alaska Native	37 (0.1)	30 (0.3)	17 (0.2)	47 (0.6)	
Asian	301 (1.0)	37 (0.4)	13 (0.1)	10 (0.1)	
Black or African American	287 (1.0)	32 (0.3)	17 (0.2)	7 (0.1)	
Native Hawaiian or Other Pacific Islander	14 (<0.1)	9 (0.1)	4 (<0.1)	3 (<0.1)	
White	27,693 (94.4)	10,119 (96.0)	8722 (96.5)	7502 (96.3)	
Other	301 (1.0)	90 (0.9)	74 (0.8)	46 (0.6)	
Unknown^b^	689 (2.3)	219 (2.1)	187 (2.1)	178 (2.3)	
Primary language					< .0001
English	28,727 (98.0)	10,364 (98.4)	8917 (98.7)	7678 (98.5)	
Other	595 (2.0)	172 (1.6)	117 (1.3)	115 (1.5)	
Service line^c^					< .0001
Medical	10,310 (35.2)	3918 (37.2)	3779 (41.8)	3073 (39.4)	
Surgical	19,012 (64.8)	6618 (62.8)	5255 (58.2)	4720 (60.6)	
Elixhauser comorbidity index	4 (0–21)	4 (0–19)	4 (0–21)	4 (0–23)	< .0001
Median length of stay, days					< .0001
≤3	18,402 (62.8)	6645 (63.1)	5553 (61.5)	4697 (60.3)	
>3	10,920 (37.2)	3891 (36.9)	3481 (38.5)	3096 (39.7)	
Median time to return survey, days					.0006
≤30	15,817 (53.9)	5476 (52.0)	4756 (52.6)	4061 (52.1)	
>30	13,505 (46.1)	5060 (48.0)	4278 (47.4)	3732 (47.9)	

Data are median (range) for age and Elixhauser comorbidity index, and frequency (%) for others.

Rurality of patients’ residence based on RUCA codes: metropolitan (codes 1–3), micropolitan (codes 4–6), small town (codes 7–9), and rural (code 10) areas.

^a^Hispanic or Latino included Central American, Cuban, Hispanic or Latino, Mexican, Puerto Rican, South American, and other Spanish culture of origin, regardless of race.

^b^Includes unknown race and chose not to disclose.

^c^Medicare Severity Diagnosis Related Groups classification.

Missing values for sex (n = 1).

*P* value from Kruskal-Wallis (age, Elixhauser comorbidity index) and Chi-square (others) tests.

Abbreviation: RUCA Rural-Urban Commuting Area.

### Patient experience

Among the survey items, *discharge information* had the highest proportion of favorable responses (range: 86.2% [rural] to 87.1% [micropolitan]; *P* = .22), followed by *recommend hospital* (range: 83.6% [small town] to 87.0% [metropolitan]; *P* < .0001) (Table 2). A high proportion of favorable responses was also observed for *communication with nurses* (range: 70.9% [small town] to 72.6% [metropolitan areas]; *P* = .02) and *communication with doctors* (range: 72.5% [rural] to 74.5% [metropolitan]; *P* = .001) (Table 2). Responses to sub-items constituting composite measures are reported in S1 and S2 Tables.

**Table 2:** Association of rurality of patients’ residence with patient experience.

	Odds ratio (95% confidence interval) for favorable vs. unfavorable response^a^					
	Composite Measures					
Rurality of Patients’ Residence	Communication with Nurses	Communication with Doctors	Responsiveness of Hospital Staff	Communication about Medicines	Discharge Information	Care Transition
Metropolitan	0.97 (0.91–1.03)	1.04 (0.97–1.10)	**0.90 (0.85–0.95)**	**0.90 (0.85–0.96)**	0.99 (0.91–1.08)	1.04 (0.98–1.10)
Micropolitan	1.00 (0.93–1.07)	1.05 (0.98–1.13)	**0.92 (0.86–0.99)**	0.96 (0.89–1.03)	1.03 (0.93–1.14)	1.01 (0.95–1.08)
Small town	0.97 (0.90–1.04)	1.03 (0.96–1.11)	0.96 (0.90–1.03)	0.97 (0.90–1.05)	1.08 (0.97–1.19)	1.04 (0.97–1.11)
vs. rural	1.00 (ref)	1.00 (ref)	1.00 (ref)	1.00 (ref)	1.00 (ref)	1.00 (ref)
*P* value (global test)	.54	.56	.002	.003	.21	.45
	**Individual Items**					
	Cleanliness of Hospital Environment			Quietness of Hospital Environment		
Metropolitan	**0.90 (0.84–0.96)**			**0.89 (0.84–0.94)**		
Micropolitan	0.99 (0.92–1.07)			**0.89 (0.83–0.95)**		
Small town	0.99 (0.92–1.08)			0.98 (0.92–1.05)		
vs. rural	1.00 (ref)			1.00 (ref)		
*P* value (global test)	< .001			< .001		
	**Global Items**					
	Overall Rating of Hospital			Recommend Hospital		
Metropolitan	**0.88 (0.81–0.94)**			0.97 (0.89–1.05)		
Micropolitan	**0.86 (0.79–0.94)**			**0.88 (0.81–0.97)**		
Small town	**0.90 (0.82–0.98)**			0.93 (0.85–1.02)		
vs. rural	1.00 (ref)			1.00 (ref)		
*P* value (global test)	.003			.04		

^a^Odds ratio (95% confidence interval) from mixed model logistic regression models adjusted for the fixed effects of age, sex (men, women), race (White, other/unknown), service line (medical, surgical), length of stay, Elixhauser comorbidity index, rurality category (metropolitan, micropolitan, small town, and rural) and random effect of study site (Rochester, Northwest Wisconsin [NWWI], Southwest Wisconsin [SWWI], Southeast Minnesota [SEMN], and Southwest Minnesota [SWMN]).

Rurality based on RUCA codes: Metropolitan (codes 1–3), micropolitan (codes 4–6), small town (codes 7–9), and rural (code 10) areas.

*P* value for global test of statistical significance across rurality categories.

Association of patient characteristics and favorable score for HCAHPS items within rurality categories is reported in S3 Table.

Abbreviations: HCAHPS Hospital Consumer Assessment of Healthcare Providers and Systems; RUCA Rural-Urban Commuting Area.

### Rurality and patient experience

The odds of favorable *overall rating of hospital* differed with rurality (global test, *P* = .003) and, compared to rural areas, was 0.88 (0.81–0.94) for metropolitan, 0.86 (0.79–0.94) for micropolitan, and 0.90 (0.82–0.98) for small town areas. For some items, the odds of a favorable response differed for select areas (e.g., *quietness of hospital environment*, and *recommend hospital*). For example, compared with rural areas, the odds of a favorable response for *recommend hospital* was lower in micropolitan (0.88 [0.81–0.97]) but not metropolitan (0.97 [0.89–1.05]) and small town (0.93 [0.85–1.02]) areas (global test, *P* = .04). For other items (e.g., *communication with nurses*, *communication with doctors*), rurality showed no association with odds of favorable response. (Table 2).

### Characteristics associated with favorable patient experience

**Fig 1:**
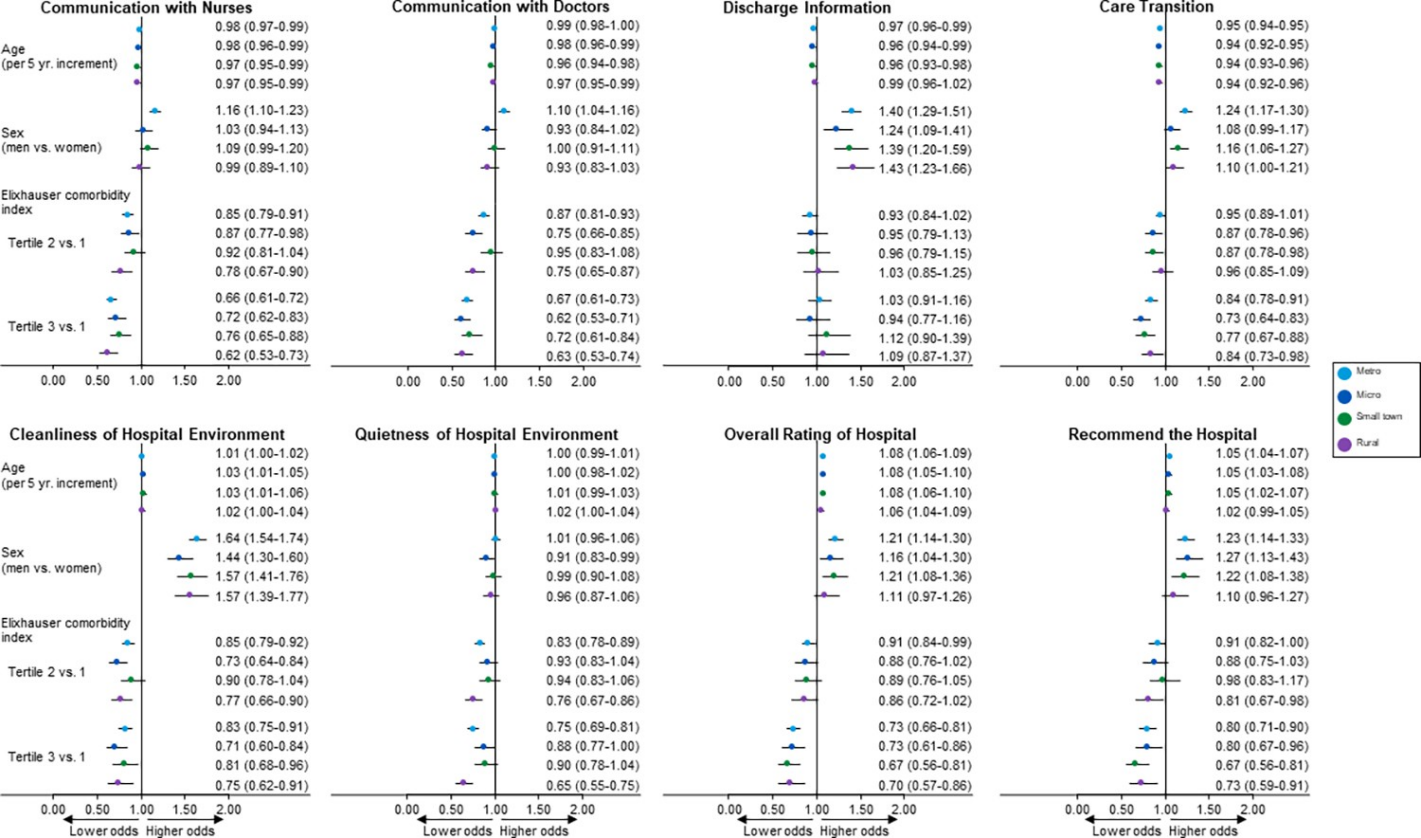
Forest plot of associations of patient characteristics and favorable patient experience scores. Adjusted odds ratio (95% confidence interval) from mixed model logistic regression models adjusted for the fixed effects of age, sex (men, women), race (White, all other), service line (medical, surgical), length of stay, Elixhauser comorbidity index, and random effect of study site (Rochester, Northwest Wisconsin [NWWI], Southwest Wisconsin [SWWI], Southeast Minnesota [SEMN], Southwest Minnesota [SWMN]). Separate logistic regression models were used for each rurality category. Two composite measures (*responsiveness of hospital staff* and *communication about medicines*) with branching logic were based on a smaller cohort of patients and are reported in S3 Table. Rurality of patients’ residence based on RUCA codes: Metropolitan (codes 1−3), micropolitan (codes 4−6), small town (codes 7−9), and rural (code 10) areas. Elixhauser comorbidity index was based on tertiles: Tertile 1 (0−2), tertile 2 (3−6), and tertile 3 (7−23). We did not compare responses across study sites to protect site confidentiality. Abbreviations: HCAHPS Hospital Consumer Assessment of Healthcare Providers and Systems; RUCA Rural-Urban Commuting Area.

In metropolitan areas, age (per 5-year increment) was associated with a higher odds of favorable response for *overall rating of hospital* (1.08 [1.06–1.09]) and *recommend hospital* (1.05 [1.04–1.07]) (Fig 1 and S3 Table). Men, compared with women, had a higher odds of favorable response for all items ranging from 1.10 (1.04–1.16) for *communication with doctors* to 1.64 (1.54–1.74) for *cleanliness of hospital environment*, except *quietness of hospital environment* (no association). In contrast, higher Elixhauser comorbidity index (tertile 3 vs. 1) was associated with a lower odds of most survey items, for example, *care transition* (0.84 [0.78–0.91]), *communication with nurses* (0.66 [0.61–0.72]) and *communication with doctors* (0.67 [0.61–0.73]).

Similarly, in micropolitan areas, age (per 5-year increment) was associated with a higher odds of favorable *overall rating of hospital* (1.08 [1.05–1.10]) and *recommend hospital* (1.05 [1.03–1.08]) (Fig 1 and S3 Table). Men, compared with women, had a higher odds of a favorable score for *discharge information* (1.24 [1.09–1.41]), *cleanliness of hospital environment* (1.44 [1.30–1.60]), *overall rating of hospital* (1.16 [1.04–1.30]) and *recommend hospital* (1.27 [1.13–1.43]). Higher Elixhauser comorbidity index (tertile 3 vs. 1) was associated with a lower odds of favorable score for *recommend hospital*.

In small towns, increasing age (per 5-year increment) was associated with a higher odds of favorable *overall rating of hospital* (1.08 [1.06–1.10]) and *recommend hospital* (1.05 [1.02–1.07]) (Fig 1 and S3 Table). Men, compared with women, had a higher odds of favorable *discharge information* (1.39 [1.20–1.59]), and *care transition* (1.16 [1.06–1.27]). Similar to metropolitan areas, higher Elixhauser comorbidity index (tertile 3 vs. 1) was associated with a lower odds of favorable *overall rating of hospital* (0.67 [0.56–0.81]) and *recommend hospital* (0.67 [0.56–0.81]).

In rural areas, increasing age (per 5-year increment) showed no association with most survey items except with *overall rating of hospital* (1.06 [1.04–1.09]), *communication with nurses* (0.97 [0.95–0.99]) and *care transition* (0.94 [0.92–0.96]) (Fig 1 and S3 Table). Men, compared with women, had a higher odds of favorable *discharge information* (1.43 [1.23–1.66]) and *cleanliness of hospital environment* (1.57 [1.39–1.77]). Higher Elixhauser comorbidity index (tertile 3 vs. 1) was associated with a lower odds of favorable score for all survey items except *communication about medicines*, *discharge information*, and *care transition* (no association).

### Key driver analysis

**Fig 2:**
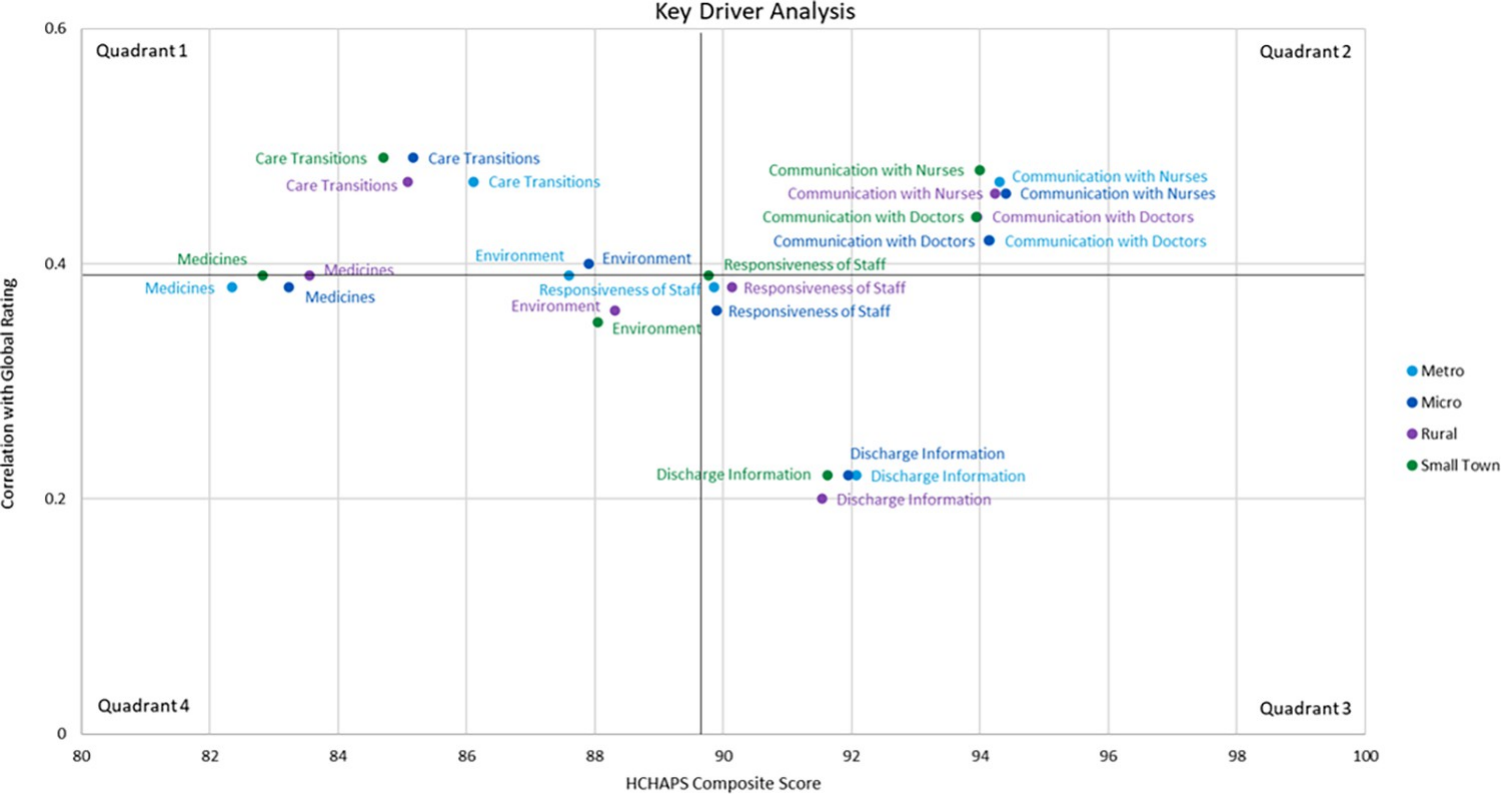
Key driver analysis of correlation between scores for HCAHPS composite measures and global rating. Quadrants 1 and 4 depict measures with relatively low composite score and high correlation (quadrant 1) or low correlation (quadrant 4) with global rating. Quadrants 2 and 3 depict measures with relatively high composite score and high correlation (quadrant 2) or low correlation (quadrant 3) with global rating. Quadrant 1 depicts measures suited for improvement. The black bold lines represent mean composite scores (vertical line) and mean global rating (horizontal line). Rurality of patients’ residence based on RUCA codes: Metropolitan (codes 1−3), micropolitan (codes 4−6), small town (codes 7−9), and rural (code 10) areas. Abbreviations: HCAHPS Hospital Consumer Assessment of Healthcare Providers and Systems; RUCA Rural-Urban Commuting Area.

The pattern of correlation between composite scores and global rating was similar across rurality categories, with slightly weaker correlations for small towns and rural areas (Fig 2). Quadrant 1 included *care transition*, which had low composite scores and high correlation with global rating, and therefore suited for improvement. Quadrants 1 and 4 included *communication about medicines* and *environment*, which had low composite scores and moderate correlation with global rating. Quadrant 2 included *communication with nurses* and *communication with doctors*, which had high composite scores and high correlation with global rating. Other items showed intermediate patterns.

## Discussion

Among 56,685 patients from an institution’s 17 hospitals in the US Midwest, favorable response to most HCAHPS items varied by rurality of patients’ residence. For *overall rating of hospital*, patients from metropolitan, micropolitan, and small towns had ~10% lower odds of favorable response compared to rural patients. For other items, patients from select areas had 5%–10% lower odds of favorable response compared to rural patients or rurality showed no association. To our knowledge, this is the first US report to characterize rurality of patients’ residence and the determinants and drivers of hospital experience. This approach may be adapted elsewhere to identify system-wide and rurality-specific opportunities to improve patient experience.

Among individual items, patients from rural vs. metropolitan areas had higher odds of favorable response for *cleanliness of hospital environment* and *quietness of hospital environment*, whereas patients from rural vs. micropolitan areas only had higher odds for the latter. Several studies have sought to improve the hospital environment using therapy dogs, sleep hygiene bundles, and flyers to explain the disinfectant used [1,22–25]. However, the studies were based on small samples, single sites, and did not stratify on rurality. Furthermore, patients may have different expectations, and interventions may be more effective if tailored to patients’ preferences.

Among composite measures, patients from rural areas (vs. metropolitan and micropolitan areas) had higher odds of favorable response for *responsiveness of hospital staff*, which was similar to results from a nationwide study of 2558 hospitals, [13] but different from a study in Massachusetts, in which rural patients had a lower likelihood of favorable response [11]. The results likely differed because of differences in study era, regions, populations, and adjusted covariates, among other factors. Systematic reviews have identified few interventions to improve responsiveness, which may require adaptation to the institutional *milieu* [1,26].

In contrast, favorable response for composite measures including *discharge information* and *care transition* showed no association with rurality. Care transition is important for improving patient experience and post-hospital outcomes but has been challenging to improve [14,18,27–29]. Most care transition interventions have aimed at improving post-hospital outcomes, primarily, 30-day hospital readmission, and have tested diverse strategies (e.g., medication reconciliation, telephone follow-up) with mixed success [29–34]. A recent study randomized general internal medicine patients to a multimodal transitional care intervention (education, follow-up with primary care provider, telephone follow-up) vs. usual care, but observed no difference in patient satisfaction or 30-day hospital readmission [30]. In a study to improve patient experience during the hospital-to-home transition, randomization to a navigator intervention (delivered by community health workers and peer coaches) vs. usual care did not improve patient experience or post-discharge outcomes [34]. Most interventions were not designed for and/or did not report effect by rurality, therefore, the potential benefit to rural populations is unknown. Furthermore, rural populations may have unique challenges (e.g., transportation between remote areas and hospital/clinic) [35], which are not captured in the HCAHPS survey. Therefore, our finding that rurality was not associated with favorable *care transition* is reassuring; however, further work is required to explore if the HCAHPS survey comprehensively evaluates experiences of rural patients.

In addition to differences across rurality, we also observed differences in the determinants of favorable response within each rurality category. In metropolitan areas, men had a higher odds of favorable response for most items, whereas in rural areas, this sex difference was largely absent. Our results on sex differences were consistent with previous non-rurality stratified studies, in which, men tended to report more favorable experiences than women [36–41]. For example, in a study at a university medical center in the Netherlands, women, particularly those aged 45–64 years and with higher education, rated their hospital experience lower than men [37]. In a study of 3122 patients from 50 US hospitals, women, compared with men, reported worse experience in the quality of emergency department care [38]. Differences from these and other studies [36–41] were attributed to sex differences in education level, comorbidity burden, preference for room privacy, and sex concordance with providers. In our institution, most rooms are single occupancy and none with more than double occupancy. Although not directly evaluated, we hypothesize that single occupancy rooms may positively affect quietness and cleanliness of the hospital room, protect confidential information discussed with physicians and nurses, and permit discussions on concerns during and after hospitalization, which may positively impact hospital experience. In our study, the sex difference persisted after adjusting for several covariates, but we did not account for patient education level or sex concordance with providers, topics that will be addressed in future work. We were intrigued by the lack of sex differences for most items in rural patients. To our knowledge, there were no rural studies on the association of sex and patient experience. The different associations (i.e., metropolitan vs. rural) suggest the need for qualitative studies to identify patient preferences or factors not captured in the HCAHPS survey.

For most items, higher comorbidity burden was associated with lower odds of favorable response, with generally similar strengths of association across rurality categories. This finding builds on results from the 2015 Chinese General Social Survey, in which rural and urban respondents with *better self-rated health* had higher odds of favorable experience [15]. Patients with a higher comorbidity burden may have higher acute care and/or care transition needs, resulting in a generally lower odds of favorable experience [42,43]. To our knowledge, there are no US reports on comorbidities, rurality, and patient experience, and the present study showed comparable experience of patients in rural and other areas.

We found it difficult to reconcile results for survey items across rurality categories. For most composite measures, rurality showed no association with favorable response, and for few items only select areas showed association. For all items (except, *overall rating of* hospital), there was no difference between small towns and rural areas. However, rural areas (vs. all areas) had a higher odds of favorable *overall rating of hospital*. This apparent discordance suggests that (i) patients from metropolitan, micropolitan, and small towns attribute different survey items to overall rating, and/or (ii) other facets of hospital experience or outcomes, not captured in the current HCAHPS survey, may inform *overall rating of hospital*. While our results for rural patients are reassuring, the survey may not capture all facets of their hospital experience. For instance, rural patients often experience more challenges with involving family members in hospital care due to long travel distance [12,44,45]. Finally, patients from metropolitan, micropolitan, small towns, and rural areas may have different expectations, as was suggested in a study on outpatient practice [16]. Qualitative studies may yield insight into the challenges and expectations of patients based on rurality of their residence.

### Strengths and limitations

Similar to other retrospective studies, our study had limitations. While the survey response rate was higher than the national average, the significance of these findings to non-respondents is unknown. We included surveys from 17 hospitals in two US states; despite the large sample size, external generalizability requires evaluation. There are several county- and subcounty- level classification systems for rurality [46]. We used one definition of rurality (i.e., RUCA) and the implication of using other definitions is not known. In future work, we will compare patient experience based on different rurality classification systems. Finally, our study included 13.7% rural respondents (n = 7793/56,685), which may not be representative of the overall rural population in these two US states. Furthermore, we did not estimate the distance travelled by rural residents to reach a hospital, which may also impact their experience, and should be the topic of future studies. Our study has strengths. To our knowledge, it is the first report on hospital experience and rurality of patients’ residence. The study investigated the key drivers of global rating and identified composite measures for improvement. The study identified potential gaps and opportunities in the current survey to comprehensively capture patient experience based on rurality.

## Conclusions

In this multisite analysis of 56,685 patients discharged from an institution’s 17 hospitals across two US states, hospital experience differed by rurality of patients’ residence. Patients in rural areas, compared with patients from other areas, had similar or more favorable hospital experience. The determinants of favorable hospital experience may guide rurality-specific and system-wide improvement. Further studies are required to explore if the current HCAHPS survey captures the unique challenges experienced by rural patients.

## Supporting information

S1 Table Patient experience for composite measures based on rurality of patients’ residence. (DOCX)

S2 Table Patient experience based on rurality of patients’ residence. (DOCX)

S3 Table Association of patient characteristics with patient experience based on rurality of patients’ residence. (DOCX)
