# Supplementary material for: Rurality and patients’ hospital experience: A multisite analysis from a US healthcare system
Source: PLoS One. 2024 Aug 8;19(8):e0308564. doi: 10.1371/journal.pone.0308564 (PMC11309381; doi:10.1371/journal.pone.0308564)
Supplement: S1 Table — (DOCX) [file pone.0308564.s001.docx]

**S1 Table: Patient Experience for Composite Measures Based on Rurality of Patients’ Residence**

|  | **Rurality of Patients’ Residence** | | | |
| --- | --- | --- | --- | --- |
|  | **Metropolitan**  **(N=29,322)** | **Micropolitan**  **(N=10,536)** | **Small town**  **(N=9034)** | **Rural**  **(N=7793)** |
| HCAHPS Composite Measures | Favorable response, no. (%) | | | |
| Communication with Nurses |  |  |  |  |
| Treated with courtesy and respect | 23,791 (91.2) | 8549 (91.3) | 7323 (90.8) | 6379 (91.5) |
| Listened carefully | 21,346 (81.9) | 7707 (82.4) | 6557 (81.5) | 5712 (82.1) |
| Explained things in a way you could understand | 20,993 (80.8) | 7491 (80.2) | 6312 (78.7) | 5523 (79.5) |
| Communication with Doctors |  |  |  |  |
| Treated with courtesy and respect | 23,747 (91.3) | 8551 (91.5) | 7340 (91.3) | 6312 (90.8) |
| Listened carefully | 21,886 (84.2) | 7890 (84.5) | 6718 (83.8) | 5827 (84.1) |
| Explained things in a way you could understand | 20,666 (79.8) | 7359 (79.0) | 6261 (78.3) | 5377 (77.7) |
| Responsiveness of Hospital Staff |  |  |  |  |
| Received help when used call button | 17,034 (71.6) | 6021 (71.8) | 5140 (70.9) | 4570 (72.5) |
| If needed, helped to use the bathroom or bedpan | 13,590 (76.0) | 5001 (76.2) | 4259 (75.2) | 3668 (76.4) |
| Communication about Medicines |  |  |  |  |
| Explained purpose of new medicine | 14,873 (80.5) | 5295 (81.0) | 4470 (80.3) | 3904 (80.9) |
| Explained side effects of new medicine | 9278 (52.6) | 3297 (53.0) | 2812 (53.3) | 2483 (54.2) |
| Discharge Information |  |  |  |  |
| Staff discussed help needed after discharge | 21,090 (91.2) | 7615 (91.2) | 6415 (90.7) | 5561 (90.8) |
| Received information on symptoms or health problems | 20,800 (93.2) | 7460 (92.8) | 6282 (92.7) | 5435 (92.4) |
| Care Transition |  |  |  |  |
| Staff considered needs after discharge | 15,171 (59.0) | 5159 (56.0) | 4411 (55.6) | 3768 (54.9) |
| Understood how to manage health | 15,937 (61.6) | 5448 (58.7) | 4497 (56.4) | 3971 (57.6) |
| Clearly understood purpose of medicines | 16,321 (63.3) | 5656 (61.2) | 4714 (59.4) | 4198 (61.2) |

Favorable response indicates the most satisfactory response: always; score of 9 or 10; yes; definitely yes; strongly agree.

Rurality of patients’ residence based on RUCA codes: metropolitan (codes 1–3), micropolitan (codes 4–6), small town (codes 7–9), and rural (code 10) areas.

Response rate differed, in particular for items with branching logic (responsiveness of hospital staff; communication about medicines).

Abbreviation: HCAHPS Hospital Consumer Assessment of Healthcare Providers and Systems; RUCA Rural-Urban Commuting Area.
