# Supplementary material for: Rurality and patients’ hospital experience: A multisite analysis from a US healthcare system
Source: PLoS One. 2024 Aug 8;19(8):e0308564. doi: 10.1371/journal.pone.0308564 (PMC11309381; doi:10.1371/journal.pone.0308564)
Supplement: S2 Table — (DOCX) [file pone.0308564.s002.docx]

**S2 Table: Patient Experience Based on Rurality of Patients’ Residence**

|  | **Rurality of Patients’ Residence** | | | |  |
| --- | --- | --- | --- | --- | --- |
|  | **Metropolitan**  **N=29,322** | **Micropolitan**  **N=10,536** | **Small town**  **N=9034** | **Rural**  **N=7793** | ***P* value** |
| HCAHPS items | Number of responses  Favorable response, no. (%) | | | |  |
| **Composite Measures** |  |  |  |  |  |
| Communication with Nurses | N=22,171  18,968 (72.6) | N=7960  6801 (72.5) | N=6684  5722 (70.9) | N=5834  5020 (71.9) | .02 |
| Communication with Doctors | N=22,675  19,403 (74.5) | N=8109  6931 (74.1) | N=6847  5860 (72.8) | N=5884  5040 (72.5) | .001 |
| Responsiveness of Hospital Staff | N=24,838  16,526 (66.5) | N=8831  5894 (66.7) | N=7654  5063 (66.1) | N=6621  4466 (67.5) | .40 |
| Communication about Medicines | N=18,554  9733 (52.5) | N=6556  3487 (53.2) | N=5588  2966 (53.1) | N=4839  2621 (54.2) | .18 |
| Discharge Information | N=26,287  20,246 (87.0) | N=9447  7329 (87.1) | N=8060  6163 (86.4) | N=6940  5314 (86.2) | .22 |
| Care Transition | N=14,762  11,414 (43.9) | N=5138  3916 (42.0) | N=4293  3269 (40.8) | N=3712  2831 (41.0) | <.0001 |
| **Individual Items** |  |  |  |  |  |
| Cleanliness of Hospital Environment | N=23,700  20,298 (78.3) | N=8665  7429 (79.9) | N=7425  6384 (79.9) | N=6411  5552 (80.1) | .0002 |
| Quietness of Hospital Environment | N=19,300  15,824 (61.2) | N=6909  5633 (60.8) | N=5917  4857 (60.9) | N=5192  4293 (62.3) | .25 |
| **Global Items** |  |  |  |  |  |
| Overall Rating of Hospital | N=25,112  21,748 (83.8) | N=8947  7738 (83.0) | N=7592  6568 (82.0) | N=6699  5819 (84.2) | .0004 |
| Recommend Hospital | N=25,951  22,517 (87.0) | N=9091  7828 (84.4) | N=7725  6685 (83.6) | N=6787  5894 (85.4) | <.0001 |

Favorable response indicates the most satisfactory response: always; score of 9 or 10; yes; definitely yes; strongly agree.

Rurality of patients’ residence based on RUCA codes: metropolitan (codes 1–3), micropolitan (codes 4–6), small town (codes 7–9), and rural

(code 10) areas.

Responses to survey items for composite measures are reported in S1 Table in the Supplement.

*P* value from Chi-Square test.

Abbreviations: HCAHPS Hospital Consumer Assessment of Healthcare Providers and Systems; RUCA Rural-Urban Commuting Area.
