# Supplementary material for: Rurality and patients’ hospital experience: A multisite analysis from a US healthcare system
Source: PLoS One. 2024 Aug 8;19(8):e0308564. doi: 10.1371/journal.pone.0308564 (PMC11309381; doi:10.1371/journal.pone.0308564)
Supplement: S3 Table — (DOCX) [file pone.0308564.s003.docx]

**S3 Table: Association of Patient Characteristics with Patient Experience Based on Rurality of Patients’ Residence**

|  | **Rurality of Patients’ Residence** | | | | |
| --- | --- | --- | --- | --- | --- |
|  | **Metropolitan** | | **Micropolitan** | **Small town** | **Rural** |
|  | Adjusted odds ratio (95% confidence interval) of favorable vs. unfavorable response^a^ | | | | |
|  | Communication with Nurses | | | | |
| Age, per 5-year increment | 0.98 (0.97–0.99)* | | 0.98 (0.96–0.99) | 0.97 (0.95–0.99)* | 0.97 (0.95–0.99)* |
| Sex, men vs. women | 1.16 (1.10–1.23)* | | 1.03 (0.94–1.13) | 1.09 (0.99–1.20) | 0.99 (0.89–1.10) |
| Elixhauser comorbidity index |  | |  |  |  |
| tertile 2 vs. tertile 1 | 0.85 (0.79–0.91)* | | 0.87 (0.77–0.98) | 0.92 (0.81–1.04) | 0.78 (0.67–0.90)* |
| tertile 3 vs. tertile 1 | 0.66 (0.61–0.72)* | | 0.72 (0.62–0.83)* | 0.76 (0.65–0.88)* | 0.62 (0.53–0.73)* |
|  | Communication with Doctors | | | | |
| Age, per 5-year increment | 0.99 (0.98–1.00) | | 0.98 (0.96–0.99) | 0.96 (0.94–0.98)* | 0.97 (0.95–0.99) |
| Sex, men vs. women | 1.10 (1.04–1.16)* | | 0.93 (0.84–1.02) | 1.00 (0.91–1.11) | 0.93 (0.83–1.03) |
| Elixhauser comorbidity index |  | |  |  |  |
| tertile 2 vs. tertile 1 | 0.87 (0.81–0.93)* | | 0.75 (0.66–0.85)* | 0.95 (0.83–1.08) | 0.75 (0.65–0.87)* |
| tertile 3 vs. tertile 1 | 0.67 (0.61–0.73)* | | 0.62 (0.53–0.71)* | 0.72 (0.61–0.84)* | 0.63 (0.53–0.74)* |
|  | Responsiveness of Hospital Staff | | | | |
| Age, per 5-year increment | 0.99 (0.98–1.00) | | 0.98 (0.97–1.00) | 1.00 (0.98–1.02) | 0.99 (0.97–1.01) |
| Sex, men vs. women | 1.20 (1.13–1.26)* | | 1.09 (1.00–1.20) | 1.17 (1.06–1.28)* | 1.03 (0.93–1.15) |
| Elixhauser comorbidity index |  | |  |  |  |
| tertile 2 vs. tertile 1 | 0.88 (0.83–0.95)* | | 0.84 (0.75–0.95)* | 0.82 (0.72–0.94)* | 0.77 (0.67–0.88)* |
| tertile 3 vs. tertile 1 | 0.70 (0.65–0.76)* | | 0.66 (0.57–0.76)* | 0.59 (0.51–0.68)* | 0.62 (0.53–0.73)* |
|  | Communication about Medicines | | | | |
| Age, per 5-year increment | 0.98 (0.97–0.99)* | | 0.97 (0.95–0.99)* | 0.96 (0.94–0.98)* | 0.96 (0.94–0.98)* |
| Sex, men vs. women | 1.46 (1.38–1.55)* | | 1.47 (1.33–1.62)* | 1.34 (1.20–1.49)* | 1.40 (1.25–1.57)* |
| Elixhauser comorbidity index |  | |  |  |  |
| tertile 2 vs. tertile 1 | 0.95 (0.89–1.02) | | 0.87 (0.76–0.98) | 1.10 (0.96–1.26) | 1.07 (0.93–1.24) |
| tertile 3 vs. tertile 1 | 0.92 (0.84–1.01) | | 0.80 (0.68–0.93)* | 0.97 (0.82–1.14) | 0.90 (0.75–1.07) |
|  | Discharge Information | | | | |
| Age, per 5-year increment | 0.97 (0.96–0.99)* | | 0.96 (0.94–0.99)* | 0.96 (0.93–0.98)* | 0.99 (0.96–1.02) |
| Sex, men vs. women | 1.40 (1.29–1.51)* | | 1.24 (1.09–1.41)* | 1.39 (1.20–1.59)* | 1.43 (1.23–1.66)* |
| Elixhauser comorbidity index |  | |  |  |  |
| tertile 2 vs. tertile 1 | 0.93 (0.84–1.02) | | 0.95 (0.79–1.13) | 0.96 (0.79–1.15) | 1.03 (0.85–1.25) |
| tertile 3 vs. tertile 1 | 1.03 (0.91–1.16) | | 0.94 (0.77–1.16) | 1.12 (0.90–1.39) | 1.09 (0.86–1.36) |
|  | Care Transition | | | | |
| Age, per 5-year increment | 0.95 (0.94–0.95)* | | 0.94 (0.92–0.95)* | 0.94 (0.93–0.96)* | 0.94 (0.92–0.96)* |
| Sex, men vs. women | 1.24 (1.17–1.30)* | | 1.08 (0.99–1.17) | 1.16 (1.06–1.27)* | 1.10 (1.00–1.21) |
| Elixhauser comorbidity index |  | |  |  |  |
| tertile 2 vs. tertile 1 | 0.95 (0.89–1.01) | | 0.87 (0.78–0.96) | 0.87 (0.78–0.98) | 0.96 (0.85–1.09) |
| tertile 3 vs. tertile 1 | 0.84 (0.78–0.91)* | | 0.73 (0.64–0.83)* | 0.77 (0.67–0.88)* | 0.84 (0.73–0.98) |
|  | Cleanliness of Hospital Environment | | | | |
| Age, per 5-year increment | 1.01 (1.00–1.02) | | 1.03 (1.01–1.05)* | 1.03 (1.01–1.06)* | 1.02 (1.00–1.04) |
| Sex, men vs. women | 1.64 (1.54–1.74)* | | 1.44 (1.30–1.60)* | 1.57 (1.41–1.76)* | 1.57 (1.39–1.77)* |
| Elixhauser comorbidity index |  | |  |  |  |
| tertile 2 vs. tertile 1 | 0.85 (0.79–0.92)* | | 0.73 (0.64–0.84)* | 0.90 (0.78–1.04) | 0.77 (0.66–0.90)* |
| tertile 3 vs. tertile 1 | 0.83 (0.75–0.91)* | | 0.71 (0.60–0.84)* | 0.81 (0.68–0.96) | 0.75 (0.62–0.91)* |
|  | Quietness of Hospital Environment | | | | |
| Age, per 5-year increment | 1.00 (0.99–1.01) | | 1.00 (0.98–1.02) | 1.01 (0.99–1.03) | 1.02 (1.00–1.04) |
| Sex, men vs. women | 1.01 (0.96–1.06) | | 0.91 (0.83–0.99) | 0.99 (0.90–1.08) | 0.96 (0.87–1.06) |
| Elixhauser comorbidity index |  | |  |  |  |
| tertile 2 vs. tertile 1 | 0.83 (0.78–0.89)* | | 0.93 (0.83–1.04) | 0.94 (0.83–1.06) | 0.76 (0.67–0.86)* |
| tertile 3 vs. tertile 1 | 0.75 (0.69–0.81)* | | 0.88 (0.77–1.00) | 0.90 (0.78–1.04) | 0.65 (0.55–0.75)* |
|  | Overall Rating of Hospital | | | | |
| Age, per 5-year increment | 1.08 (1.06–1.09)* | | 1.08 (1.05–1.10)* | 1.08 (1.06–1.10)* | 1.06 (1.04–1.09)* |
| Sex, men vs. women | 1.21 (1.14–1.30)* | | 1.16 (1.04–1.30) | 1.21 (1.08–1.36)* | 1.11 (0.97–1.26) |
| Elixhauser comorbidity index |  | |  |  |  |
| tertile 2 vs. tertile 1 | 0.91 (0.84–0.99) | | 0.88 (0.76–1.02) | 0.89 (0.76–1.05) | 0.86 (0.72–1.02) |
| tertile 3 vs. tertile 1 | 0.73 (0.66–0.81)* | | 0.73 (0.61–0.86)* | 0.67 (0.56–0.81)* | 0.70 (0.57–0.86)* |
|  | Recommend Hospital | | | | |
| Age, per 5-year increment | 1.05 (1.04–1.07)* | 1.05 (1.03–1.08)* | | 1.05 (1.02–1.07)* | 1.02 (0.99–1.05) |
| Sex, men vs. women | 1.23 (1.14–1.33)* | 1.27 (1.13–1.43)* | | 1.22 (1.08–1.38)* | 1.10 (0.96–1.27) |
| Elixhauser comorbidity index |  |  | |  |  |
| tertile 2 vs. tertile 1 | 0.91 (0.82–1.00) | 0.88 (0.75–1.03) | | 0.98 (0.83–1.17) | 0.81 (0.67–0.98) |
| tertile 3 vs. tertile 1 | 0.80 (0.71–0.90)* | 0.80 (0.67–0.96) | | 0.67 (0.56–0.81)* | 0.73 (0.59–0.91)* |

^a^Odds ratio (95% confidence interval) from mixed model logistic regression models adjusted for the fixed effects age, sex (men, women), race (White, all other), service line

(medical, surgical), length of stay, Elixhauser comorbidity index, and random effect of study site (Rochester, Northwest Wisconsin, Southwest Wisconsin, Southeast Minnesota,

and Southwest Minnesota). Separate regression models were used for each rurality category.

**P*<.005 based on Bonferroni correction for 10 survey items.

Rurality of patients’ residence based on RUCA codes: metropolitan (codes 1–3), micropolitan (codes 4–6), small town (codes 7–9), and rural (code 10) areas.

Elixhauser comorbidity index tertiles: tertile 1 (0–2), tertile 2 (3–6), and tertile 3 (7–23).

Association of rurality of patients’ resident and HCAHPS items is reported in Table 2.

Abbreviations: HCAHPS Hospital Consumer Assessment of Healthcare Providers and Systems; RUCA Rural-Urban Commuting Area.
